# The hand tremor spectrum is modified by the inertial sensor mass during lightweight wearable and smartphone-based assessment in healthy young subjects

**DOI:** 10.1038/s41598-022-21310-4

**Published:** 2022-10-07

**Authors:** Patrícia Seixas Alves Santos, Enzo Gabriel Rocha Santos, Luis Carlos Pereira Monteiro, Bruno Lopes Santos-Lobato, Gustavo Henrique Lima Pinto, Anderson Belgamo, André Santos Cabral, Anselmo de Athayde Costa e Silva, Bianca Callegari, Givago Silva Souza

**Affiliations:** 1 Instituto de Ciências Biológicas, Universidade Federal do Pará, Belém, Brazil; 2 Instituto de Ciências Exatas e Naturais, Universidade Federal do Pará, Belém, Brazil; 3 Instituto de Ciências da Saúde, Universidade Federal do Pará, Belém, Brazil; 4 Departamento de Ciência da Computação, Instituto Federal de São Paulo, Piracicaba, Brazil; 5 Centro de Ciências Biológicas e da Saúde, Universidade do Estado do Pará, Belém, Brazil; 6 Programa de Pós-Graduação em Ciências do Movimento Humano, Instituto de Ciências da Saúde, Universidade Federal do Pará, Belém, Brazil; 7 Laboratório de Estudos da Motricidade Humana, Instituto de Ciências da Saúde, Universidade Federal do Pará, Belém, Brazil; 8 Núcleo de Medicina Tropical, Universidade Federal do Pará, Av Generalíssimo Deodoro 92, Umarizal, Belém, Pará 66055240 Brazil

**Keywords:** Health care, Neurological manifestations, Neurophysiology

## Abstract

Tremors are common disorders characterized by an involuntary and relatively rhythmic oscillation that can occur in any part of the body and may be physiological or associated with some pathological condition. It is known that the mass loading can change the power spectral distribution of the tremor. Nowadays, many instruments have been used in the evaluation of tremors with bult-in inertial sensors, such as smartphones and wearables, which can significantly differ in the device mass. The aim of this study was to compare the quantification of hand tremor using Fourier spectral techniques obtained from readings of accelerometers built-in a lightweight handheld device and a commercial smartphone in healthy young subjects. We recruited 28 healthy right-handed subjects with ages ranging from 18 to 40 years. We tested hand tremors at rest and postural conditions using lightweight wearable device (5.7 g) and smartphone (169 g). Comparing both devices at resting tremor, we found with smartphone the power distribution of peak ranging 5 and 12 Hz in both hands. With wearable, the result was similar but less evident. When comparing both devices in postural tremor, there were significant differences in both frequency ranges in peak frequency and peak amplitude in both hands. Our main findings show that in resting condition the hand tremor spectrum had a higher peak amplitude in the 5–12 Hz range when the tremor was recorded with smartphones, and in postural condition there was a significantly (*p* < 0.05) higher peak power spectrum and peak frequency in the dominant hand tremors recorded with smartphones compared to those obtained with lightweight wearable device. Devices having different masses can alter the features of the hand tremor spectrum and their mutual comparisons can be prejudiced.

## Introduction

Tremor is an involuntary, relatively rhythmic oscillation that occurs anywhere in the body^1^. It is considered common disorder^2^ and is typically the most recurrent symptoms of movement disorders^3^. In summary, there are two types of tremors: normal tremors inherent in the physiological behavior of the human body and abnormal tremors that are commonly related to certain pathological conditions^4^, such as Parkinson's disease (PD) and essential tremor (ET)^5,6^.

Previous studies investigated these tremors by recording their presence during rest and postural conditions^7,8^; and provided important differentiation tools for clinical purposes^7,9^. While resting tremors appear when the hand is relaxed, without intentional action of the muscles^10^, postural tremor occurs through voluntary contraction of the muscles to maintain the position against gravity^4^.

There is no gold standard assessment method for identifying tremors^11^ and many methods have been used in the literature, such as electromyography, video recordings, and inertial sensor recordings^12–22^.

Inertial sensors are practical, light, highly precise, and highly efficient^23^. They generate a large and detailed amount of data ready to be statistically analyzed from clinical or laboratory practices in patients experiencing motor disorders. Inertial sensors, such as accelerometers and gyroscopes, are included in wearable devices and smartphones, and many applications of these instruments have been developed to quantify tremors. Table 1 shows some references that recorded the tremor using accelerometers built-in portable devices.

**Table 1:** List of references that recorded the tremor using accelerometers built-in portable devices.

Reference	Inertial device	Population
Salarian et al.^17^	Wearable device with gyroscope	Patients with Parkinson’s disease and healthy subjects
Elble^24^	Wearable device with accelerometer	Healthy subjects
Dai et al.^19^	Wearable device with accelerometer and gyroscope	Patients with Parkinson’s disease and healthy subjects
Araujo et al.^25^	Wearable device with accelerometer and gyroscope	Healthy and Patients with Parkinson’s disease
Channa et al.^26^	Wearable device with accelerometer and gyroscope	Patients with Parkinson’s disease and healthy subjects
Kostikis et al.^27^	Smartphone with accelerometer and gyroscope	Patients with Parkinson’s disease and healthy subjects
Fraiwan et al.^28^	Smartphone with accelerometer	Patients with Parkinson’s disease and healthy subjects
Calvo & Ferrara^29^	Smartphone with accelerometer	Adults with leg tremors while standing or unsteadiness when standing

Previous studies have reported that mass loading at the location where the tremor is measured affects the spectral distribution of the tremor power^4,30–32^. The spectral peak frequency of the tremor has a linear relationship with the reciprocal of the square root of the added mass. This information led us to question the similarities between the quantification of tremors using smartphones and wearable devices. Usually, wearable devices weigh several grams while smartphones weigh hundreds of grams. Our hypothesis is that this difference in their mass can potentially lead to misclassifications of the tremor.

Thus, in the present investigation, we aimed to compare the quantification of hand tremor using Fourier spectral techniques obtained from a lightweight portable device and a commercial smartphone in healthy young subjects.

## Methods

### Ethical considerations

All subjects present in this study voluntarily agreed to participate with informed and written consent. All procedures were approved by the Research and Ethics Committee of the Federal University of Pará (Report #1.338.241). We confirm that all research was performed in accordance with Declaration of Helsinki, and informed consent was obtained from all participants.

### Subjects

The study was carried out with 28 participants (15 men, 13 women, 28.68 ± 6.05 years old) recruited for convenience. The laterality of the participants was established according to the short-form version of the Edinburgh Handedness Inventory^33^ (Veale et al., 2014). None participant reported the correction in the use of the dominant hand during the childhood. All participants were young, healthy, and right-handed adults aged 18–40 years. None of the participants had a history of neurological or systemic diseases, self-reported hand tremors that made it difficult to carry out daily activities or used any medication that caused tremors.

### Experimental procedure

**Figure 1:**
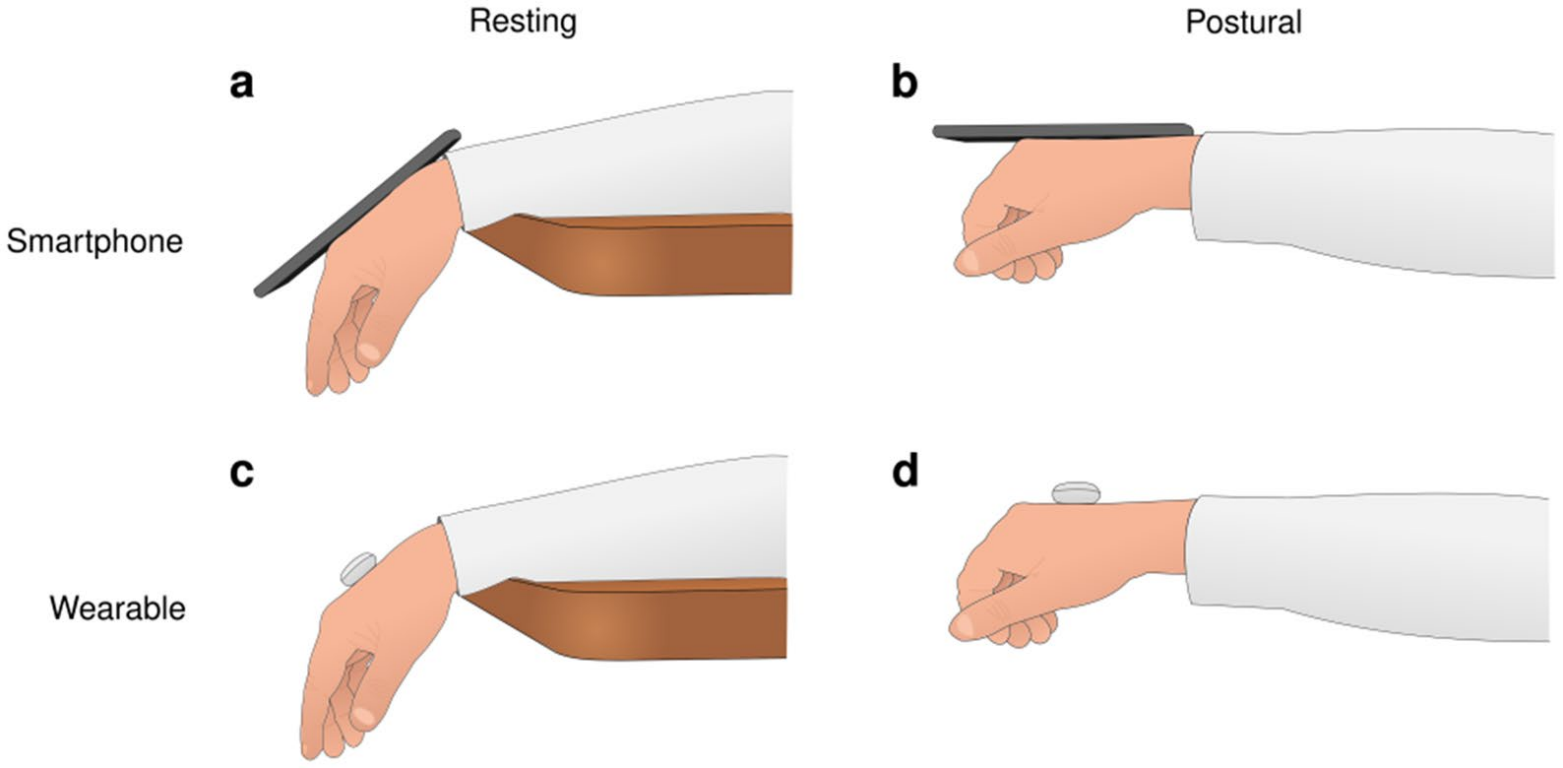
Positioning of the portable devices (lightweight wearable and smartphone) for hand tremor in resting and postural conditions. (**a**) Resting condition and smartphone. (**b**) Postural condition and smartphone. (**c**) Resting condition and lightweight wearable. (**d**) Postural condition and lightweight wearable. The devices were fixed to the hand by a double-sided tape.

Hand tremors were recorded using wearable device and smartphone (Fig. 1). The wearable device was a MetaMotionC (mbientlab, San Francisco, United States) with a 25 mm diameter, 5.7 g. This device has on-board sensors, including a triple-axis accelerometer (Bosch model, 16 bits, ± 16 g). An Android app (MetaBase, mbientlab, United States) controlled the sensors via Bluetooth using a smartphone to start and end the records, as well as to transfer digital data via Bluetooth. The acquisition frequency of the inertial sensors in the wearable device was set as 100 Hz. The smartphone was a Samsung Galaxy A20 model, 158.4 × 74.7 × 7.8 mm, with 169 g. An Android app Momentum^34,35^ was used to start and finish the smartphone sensor recording. The acquisition frequency of the accelerometer (LMS6DSL model, 16 bits, range: ± 4 g) on the mobile device was 50 Hz.

Hand tremor was recorded with both devices under four conditions: dominant hand and resting position, dominant hand and postural position, nondominant hand and resting position, and dominant hand and postural position. To perform the recording, the participant was asked to sit upright in a chair with the side of the body to be tested sitting parallel to a table (at a comfortable height) to allow the arm to relax between tests. The resting position consisted of keeping the hand off the table in a hanging position. To record the tremor in the postural position, the participant was asked to keep their fingers relaxed in the neutral position, the wrist in the neutral position, elbow fully extended, and the shoulder in a 90° flexion and 0° abduction position. The smartphone and the wearable device were fixed in the dorsal region of the hand, in the middle of the third metacarpal, between the carpus and the digital ends of the metacarpal, using double-sided tape. Figure 1 shows the placement of the sensors in each posture of the hand. The test lasted one minute for each condition, with a minimum interval of one minute between them. A record was made for each hand in different positions and devices, totaling eight records (2 hands × 2 devices × 2 positions). A random sequence was used to record tremors under different conditions for each subject.

### Data analysis

Scripts programmed in the MATLAB/Octave language (GNU Octave, version 6.3.0, 2021) to process the inertial signals obtained by the devices. The first and last 5 s of the inertial recordings were excluded from the analysis. The inertial signals were subjected to a detrend protocol to remove the effect of linear trends on the time series. Subsequently, the time series were submitted to a second-order zero-lag Butterworth filter, bandpass between 0.1 and 20 Hz. To extract features in the frequency domain, a fast Fourier transform (FFT) was applied to estimate the peak frequency of the spectrum and the peak amplitude in the ranges of 0.1–5 Hz and 5–12 Hz. A paired Student’s *t*-test was used to compare the peak frequency and peak amplitude of hand tremors using both ranges of frequency of each device. The effect size was calculated using Cohen’s *d* and was considered the Cohen effect size classification as ignored (d ≤ 0.2), small (d ˃ 0.2 and d ≤ 0.5), and medium (d ˃ 0.5 and d ≤ 0.8), and large (d > 0.8)*.* SPSS software was used for statistical analysis. A level of significance of 0.05 was considered for all analyses.

## Results

### Comparison of resting tremor evaluated by wearable and smartphone devices

**Figure 2:**
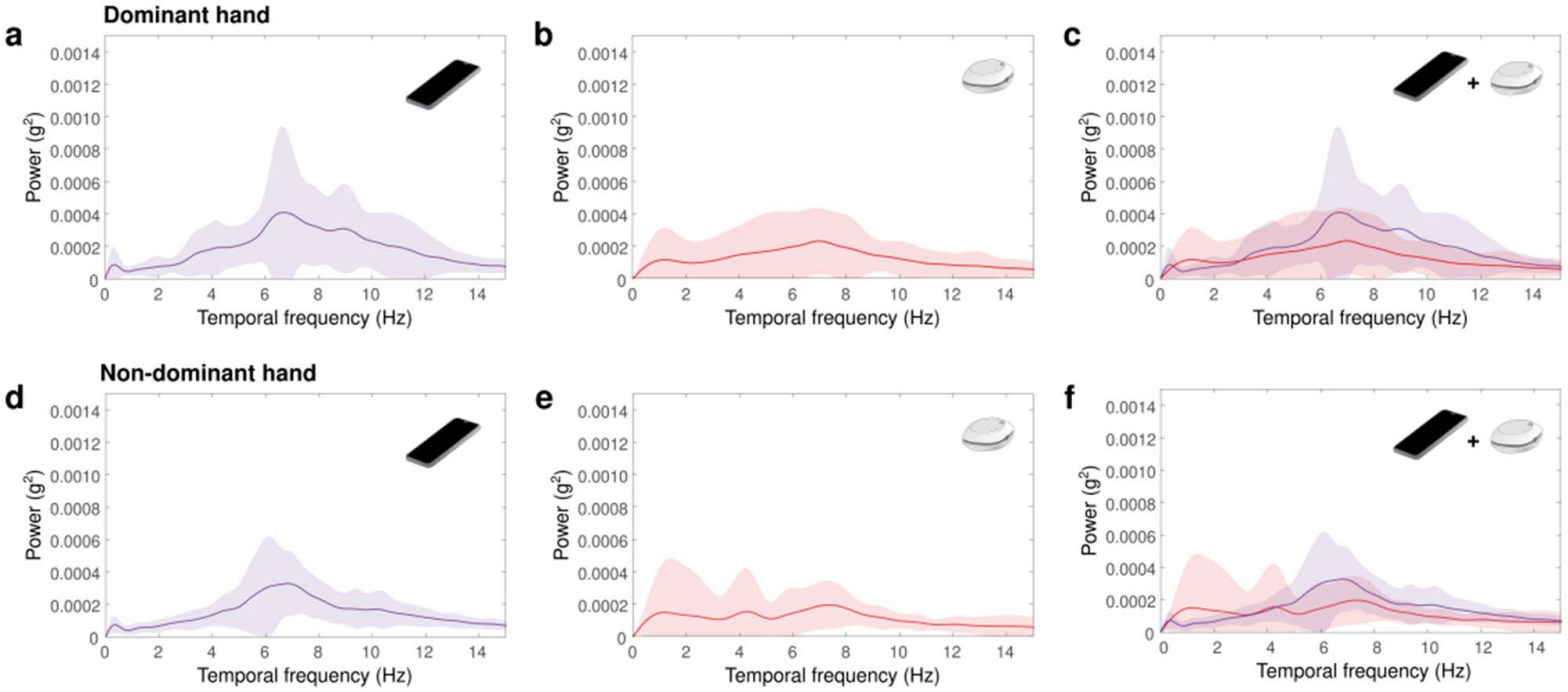
Power spectrum of tremor at rest recorded by smartphone in the dominant hand (**a**), lightweight wearable in the dominant hand (**b**), and comparison between both devices in the dominant hand (**c**). (**d**), (**e**) and (**f**) represent similar plots for non-dominant hand recordings.

The mean and dispersion (1 standard deviation) of the spectrum of the tremor of the resting hand (of the dominant and non-dominant hands) recorded by wearable and smartphone devices are shown in Fig. 2. For the recordings obtained from smartphones, the power distribution peaked ranging between 5 and 12 Hz in both hands. Similar but less conclusive results were observed for wearable devices. Table 2 shows the quantification of the tremor for both hands in the frequency ranges of 0–5 Hz and 5–12 Hz. For the non-dominant hand, a significant difference was found in the range of 0–5 Hz, in which the smartphone spectrum peaked at higher frequencies (mean ± standard deviation, SD = 4.38 ± 1.2) than those from recordings obtained using wearable devices (mean ± SD = 2.89 ± 1.8, *p* < 0.001, Cohen’s *d* = 0.97), and another significant difference was observed in the peak amplitude at the range 5–12 Hz, in which spectrum of the tremor obtained using smartphone (mean ± SD = 44.37 ± 30.3) had higher amplitudes than that obtained using the wearable (mean ± SD = 24.42 ± 18.4, *p* = 0.006, Cohen’s *d* = 0.8). For the dominant hand, we found a significant difference in the peak amplitude in the range of 5–12 Hz, in which the peak amplitude obtained with the smartphone (mean ± SD = 56.85 ± 54.2) was greater than that obtained with the wearable device (mean ± SD = 27.97 ± 25, *p* = 0.008, Cohen’s *d* = 0.68).

**Table 2:** Comparison of the features of the tremors of the resting hand (mean ± SD) obtained using wearable and smartphone devices.

Parameters	Wearable	Smartphone	t-test *p* value
**0–5 Hz**			
*Dominant hand*			
Peak amplitude	19.02 ± 25.9	27.1 ± 19.4	0.15
Peak frequency	4.31 ± 0.9	4.38 ± 1	0.69
*Non-dominant hand*			
Peak amplitude	19.47 ± 34.7	21.4 ± 13.9	0.7
Peak frequency	2.89 ± 1.8	4.38 ± 1.2	< 0.001*
**5–12 Hz**			
*Dominant hand*			
Peak amplitude	27.97 ± 25	56.85 ± 54.2	0.008*
Peak frequency	7.41 ± 1.42	7.26 ± 1.38	0.63
*Non-dominant hand*			
Peak amplitude	24.42 ± 18.4	44.37 ± 30.3	0.006*
Peak frequency	7.81 ± 1.09	7.16 ± 1.51	0.08

*Significant difference using the paired-t test.

### Comparison of postural tremor evaluated by wearable and smartphone devices

**Figure 3:**
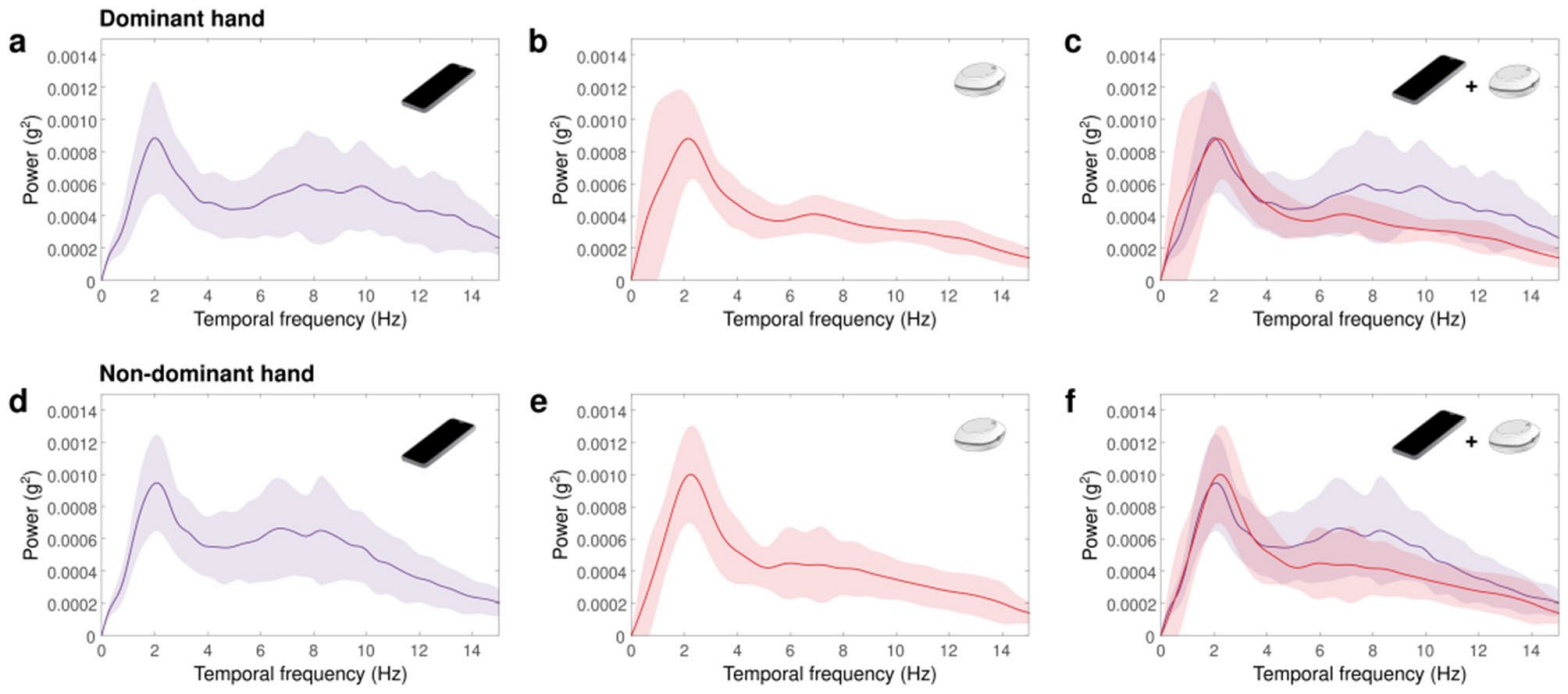
Power spectrum of the tremor in postural condition recorded by the smartphone in the dominant hand (**a**), lightweight portable in the dominant hand (**b**), and comparison between both devices in the dominant hand (**c**). (**d**), (**e**) and (**f**) represent similar plots for non-dominant hand recordings.

Figure 3 shows the power distribution as a function of the temporal frequency of the postural tremor recordings obtained using both devices. For the smartphone spectrum, it was clearly observed that there were two peaks, one in the low-frequency range (0–5 Hz) and a second peak in the high-frequency range (5–12 Hz). In the case of the wearable device, the peak of the low-frequency range was easily found, but the peak in the high-frequency range was not clearly visible. Table 3 shows the comparisons between the quantification of tremors obtained using both devices under postural conditions. In the low-frequency range, it was observed that smartphones and wearable devices had a similar peak amplitude in the dominant hand. The main differences were found in the high-frequency range, which we observed that, for the dominant hand, smartphone recordings had a larger peak amplitude (mean ± SD = 82.28 ± 37.7) and higher peak frequency (mean ± SD = 8.31 ± 1.96) than recordings obtained using wearable devices (peak amplitude: mean ± SD = 47.82 ± 11.2, *p* < 0.001, Cohen’s *d* = 1.24; peak frequency: mean ± SD = 7.06 ± 1.92; *p* = 0.007, Cohen’s *d* = 0.64). Regarding the nondominant hand, the smartphone recordings had a larger peak amplitude (mean ± SD = 89.13 ± 35.3) than the recordings from the wearable devices (mean ± SD = 53.29 ± 22.1, *p* < 0.001, Cohen’s *d* = 1.21).

**Table 3:** Comparison of the postural hand tremor (mean ± SD) obtained using wearable and smartphone devices.

Parameters	Wearable	Smartphone	*p* value
**0–5 Hz**			
*Dominant hand*			
Peak amplitude	97.33 ± 43.1	94.39 ± 34.2	0.71
Peak frequency	2.12 ± 0.5	2.01 ± 0.4	0.12
*Non-dominant hand*			
Peak amplitude	103.36 ± 31.4	101.74 ± 28.7	0.73
Peak frequency	2.23 ± 0.23	2.14 ± 0.64	0.43
**5–12 Hz**			
*Dominant hand*			
Peak amplitude	47.82 ± 11.2	82.28 ± 37.7	< 0.001*
Peak frequency	7.06 ± 1.92	8.31 ± 1.96	0.007*
*Non-dominant hand*			
Peak amplitude	53.29 ± 22.1	89.13 ± 35.3	< 0.001*
Peak frequency	7.44 ± 2.1	7.66 ± 1.8	0.67

## Discussion

The motivation for this research is to assess whether the addition of devices with different masses, such as smartphones and lightweight wearable devices, would modify the characteristics of inertial recording of tremors, as observed in previous studies that investigated the hand and fingers^24,30,31^. Our main findings were that the tremor spectrum obtained from both devices differed depending on the spectrum range, handedness, and position of the segment during testing. These results have significant implications for the comparison of tremors obtained using smartphones and wearable devices.

It is well known that mass addition to the segment to be recorded leads to a change in the acceleration power distribution of the tremor spectrum^24,30,31,36–38^. In summary, the hand tremor had a single peak without additional mass, and with the addition of mass, most participants had tremors with a double-peaked spectrum (with little or no influence of the mass loading on the tremor amplitude). Our hypothesis is that with the use of smartphones (devices with a dozen of grams), participants would experience conditions that are more similar to the experimental conditions of mass loading than during the use of the lightweight wearable device (devices with few grams). Furthermore, the tremors obtained using smartphones would present changes similar to those observed in the experiments with mass loading.

Our findings partially confirmed our expectations. Under the rest conditions, we observed a numerical and nonsignificant reduction of the spectral peak in the range of 5–12 Hz for the recordings obtained using a smartphone compared to wearable devices for both hands. It was found a reduction of 2 Hz in the spectral peak after a 300 g loading^24^. In the present study, the smartphone had approximately 169 g and was associated with spectral peak reduction of a few units of frequency, which was expected to be smaller than that reported previously^24^. We also observed that with smartphone loading, especially in the dominant hand, the mean spectrum was double-peaked, with one component peaking in the range between 6 and 8 Hz, and another component peaking in the range between 8 and 10 Hz. On the contrary, while using lightweight wearable devices, the tremor was characterized by a single peak in the range between 6 and 8 Hz. In the low-frequency range, we observed a higher peak frequency for the smartphone spectrum than that for the wearable spectrum. These findings likely reflect the spread of the high energy found in the high-frequency range observed in the spectra of the smartphone.

During postural conditions, the tremor we recorded probably involved a mixture of tremors from different parts of the upper limb. We observed a narrow power distribution in the low frequency range, likely representing the tremor of the proximal parts of the upper limb, in addition to a broad power distribution in the high frequency range, representing the tremor of the distal parts of the upper limb. The main difference between the tremor spectra obtained from the smartphone and the wearable devices resided in the power distribution in the high frequency range. There were no significant differences between devices between the peak power and peak frequency in the low-frequency range; however, the peak power in the high-frequency range was larger in the tremor spectrum from the smartphone recordings than in the wearable recordings for both hands. Furthermore, we found a significantly higher peak frequency for smartphones than for wearables on the dominant and non-dominant hands.

The mass of the device during a hand tremor test has previously been discussed as a possible response to the results found in the extended posture test, particularly in comparison with the dominant and nondominant hands^27,39^. Some authors have investigated tremors with portable, noncontact tremor vibration measurements in the hand^40^. Considering various multiple devices that are commercially available, the different weights they can present and the possible change in the signal spectrum they can cause, it would be interesting to inform the mass of the devices in the investigation of body tremors.

The present study had limitations with respect to the number of devices with different masses used to quantify tremors. For future investigations, it would be of great value to perform several measurements of limb tremor with several smartphones or wearable devices (with different masses) in order to compare possible differences in the spectral characteristics of their signals. The generalizability of the present study was also limited by the age range of our sample comprised by young healthy subjects. Physiological tremor, especially in the postural component, has been shown to be affected by aging and by chronic non-neurological conditions that are very common in the general population (e.g., hypertension)^41^. Furthermore, no participant had any disease in our experiments and future investigation with patients suffering from different diseases that affected the hand tremor is necessary.

In the present investigation, we placed the devices in the dorsum of the hand. Although it has been previously used, this placement is quite acceptable for assessments in the laboratory or, to a lesser extent, at home, but not for long-term daily recordings. For long-term recordings of tremor, it is reasonable to explore the promising potentialities of wearable inertial measurement units and the placement in the different body segments of the patients.

Our investigation has no information about basal condition (without additional mass) of physiologic tremor. We considered that the few grams of the lightweight wearable device could approximate this basal condition. A more controlled approach could use an electromyographic recordings associated to the inertial recordings during no mass loading condition, lightweight wearable loading, and smartphone loading.

Many clinical investigations have reported the importance of mass loading for the evaluation of essential tremor^42,43^. Essential tremor has a similar power distribution compared to the physiological tremor represented by a mechanistic component at 5–12 Hz^31^. However, as previously discussed, the mass loading decreases the maximum power of the mechanical resonance of the tremor, allowing identification of the neural component of the essential tremor^40^.

Thus, the use of a device with higher mass, such as smartphones, could improve the correct identification of neurological tremors. The use of built-in accelerometers inside portable devices, such as smartphones and wearable devices, opens a new window of opportunity for a low-cost and objective evaluation of physiological and pathological tremors for people with different social conditions and can be an important tool for health care in the future. Comprehension of the evaluation results obtained using these different portable devices is necessary to improve their validity for clinical use.

## Data Availability

The datasets generated during and/or analyzed during the current study are available from the corresponding author on reasonable request.
